# Resistant ovary syndrome: Two case reports and a literature review of effective controlled ovarian stimulation in IVF

**DOI:** 10.1097/MD.0000000000037886

**Published:** 2024-05-03

**Authors:** Shanfei Zhao, Wenling Zheng, Xinru Gu, Guanglin Liang, Guanyun Long

**Affiliations:** a Reproductive Medicine Center, Department of Obstetrics and Gynecology, Maoming People’s Hospital, Maoming, Guangdong, China.

**Keywords:** case report, controlled ovarian stimulation, conventional in vitro fertilization, pituitary downregulation, resistant ovary syndrome

## Abstract

**Introduction:**

Resistant ovary syndrome (ROS) represents a rare reproductive endocrine disorder that is predominantly associated with infertility, characterized by heightened endogenous gonadotropin levels in the presence of a normal ovarian reserve. Patients with ROS typically exhibit a poor response to exogenous gonadotropins during controlled ovarian stimulation (COS). Due to the absence of a universally accepted effective COS protocol, this study aims to contribute to the existing body of literature by detailing 2 successful pregnancies achieved through conventional in vitro fertilization (c-IVF) in patients with ROS, and through retrospective analysis, seeks to elucidate the factors contributing to the successful ovarian stimulation in these cases, with the ultimate goal of establishing clinical guidelines for ROS management.

**Patient Concerns:**

The central challenge addressed in this study pertains to the effective induction of oocyte maturation during c-IVF COS in ROS patients.

**Diagnosis:**

The study focuses on 2 infertile women diagnosed with ROS who sought to conceive via c-IVF.

**Interventions:**

The patients were subjected to a COS protocol involving pituitary downregulation followed by ovarian stimulation using recombinant follicle-stimulating hormone (r-FSH) and human menopausal gonadotropin (HMG), preceded by 3 cycles of hormone replacement therapy (HRT) pretreatment.

**Outcomes:**

The proposed protocol elicited a favorable ovarian response, culminating in the retrieval of numerous mature oocytes and the development of multiple viable embryos via c-IVF, ultimately leading to successful live births post-embryo transfer.

**Conclusions:**

Our study suggests that the outlined COS protocol may serve as a viable treatment option for ROS patients aspiring to conceive through c-IVF, thereby potentially expanding the therapeutic repertoire for this challenging condition.

## 1. Introduction

Resistant ovary syndrome (ROS), also known as Savage syndrome, was first proposed by Jones and Ruehsen in 1969.^[1]^ It is a reproductive endocrine disorder with a low incidence and diagnostic rate. It is characterized by elevated endogenous gonadotropin (Gn) and unresponsiveness to large doses of exogenous Gn. It is distinct from irreversible premature ovarian failure (POF), as it is demonstrated by normal antral follicle counts and age-appropriate serum anti-Müllerian hormone (AMH).^[1–4]^ Patients with ROS often present with primary or secondary amenorrhea, although they have normal sexual characteristics and karyotype.^[1–4]^

Due to the low incidence of ROS, few large sample studies have been conducted involving successful cases. Pregnancies resulting from the patient’s own oocytes have mainly been achieved through hormone replacement therapy (HRT)^[5–12]^ or in vitro maturation (IVM),^[13–19]^ and a handful of conventional in vitro fertilization (c-IVF) after controlled ovarian stimulation (COS).^[17,20–22]^ Therefore, ROS patients who are unable to produce mature oocytes may have to resort to donor oocytes.^[23–25]^ While IVM and c-IVF enable ROS patients to obtain genetically related offspring, which is more acceptable than donor oocytes, most reproductive centers cannot carry out IVM and it is relatively expensive, so c-IVF remains an important means of assisted fertility for ROS patients. At present, there is no clear consensus on the regimens of COS for ROS patients. Here, we report 2 cases of patients diagnosed with ROS, who achieved clinical pregnancy through c-IVF. These 2 cases are interesting as they underwent the same protocol for COS and successfully achieved pregnancy, a feat that has yet to be achieved.

## 2. Case presentation

This study has been officially approved by the Clinical Research Review Committee of Maoming People’s Hospital. Written informed consent was obtained from the patient and her family for the publication of this case report.

### 2.1. Case 1

#### 2.1.1. Patient history

In August 2015, a 25-year-old woman visited our IVF center due to primary infertility over the past 3 years. Her menstruation cycle had been irregular since menarche at 14 with a cycle of 60 to 120 days. She had been diagnosed with polycystic ovary syndrome at another hospital and had occasionally taken oral contraceptives to induce menses. A diagnostic laparoscopic exploration revealed a slight tubal adhesion and enlarged ovarian appearance, but no other pelvic lesions. She failed to respond to oral ovulation stimulation drugs. The patient had normal secondary sexual characteristics and a low body mass index (BMI) of 15.6, and showed no clinical signs of hyperandrogenemia or other pituitary dysfunction. Her karyotype was 46, XX. The present study was conducted in accordance with the ethical guidelines of the affiliated institution, and the patient provided informed consent.

#### 2.1.2. Hormone measurements and ultrasound scans

Initial hormone measurements and ultrasound scanning were performed in a state of amenorrhea. A chemiluminescence automatic multi-analysis system (Roche, Switzerland) was used to determine serum FSH, luteinizing hormone (LH), estradiol (E2), prolactin, total testosterone, anti-mullerian hormone (AMH), and thyroid-stimulating hormone (TSH) levels. Ultrasound scans were performed with a 5.0 to 8.0 mhz multifrequency transvaginal probe (Aloka, Japan).

The results of the tests revealed abnormally elevated FSH and LH levels, while the AMH level was considered normal. Ultrasound scanning revealed that the uterus and ovaries were of regular size and that the ovaries contained a total of 22 antral follicles. The patient was diagnosed with ROS and was prescribed HRT (Femonton, Abbott Biologicals B.V., Netherlands), which resulted in regular withdrawal bleeding for 2 months. During this period, no evidence of follicle growth was observed in the continuous ultrasound monitoring. Hormone and ultrasonographic measurements were repeated on day 3 of the second cycle of withdrawal bleeding, which still showed high levels of FSH and LH (see Table 1 for detailed descriptions of the hormones and ultrasound scan information).

**Table 1:** Hormonal and ultrasonographic profiles.

Variable	Case 1		Case 2	
	August 21, 2015	October 16, 2015	June 17, 2020	August 12, 2020
FSH, mUI/mL	27.99	18.87	18.55	17.79
LH, mUI/mL	41.79	36.23	17.27	18.83
E2, pg/mL	143.3	56.17	38.24	37.58
T, ng/mL	0.33	–	0.45	–
P, ng/mL	0.54	–	0.12	–
PRL, ng/mL	6.95	–	14.64	–
TSH, mU/L	3.96	–	3.27	–
AMH, ng/mL	5.1	–	3.95	–
Antral follicle count, 2–9 mm	22	19	>24	>24

Note: AMH = anti-Müllerian hormone, E2 = estradiol, FSH = follicle-stimulating hormone, LH = luteinizing hormone, P = progesterone, PRL = prolactin, T = total testosterone, TSH = thyroid-stimulating hormone.

The levels of serum anti-ovarian antibody and antizona pellucida antibody were measured by ELISA (LIANER, China), which showed no abnormalities.

#### 2.1.3. Controlled ovarian stimulation for IVF

Between April 26, 2016, and January 14, 2017, the patient received 3 cycles of COS (Table 2). The first cycle (April 26, 2016) began with 225 IU/d of hMG (Lizho Pharmaceuticals, China) for 7 days on day 3 of the menstrual cycle. The dose was then increased to 300 IU/d for 6 days due to the slow development of follicles. A GnRH (Gonadotropin releasing hormone) antagonist (Cetrotide, Baxter Oncology GmbH, Germany) was administered at a dosage of 0.25 mg when the dominant follicle reached 12 mm in diameter. However, on the 14th day of ovarian stimulation, the estrogen level stopped increasing and only 2 follicles reached 18 mm in diameter, while the remaining ones were poorly developed. As a result, 10000, IU of human chorionic gonadotropin (HCG) (Lizho Pharmaceuticals) was used to trigger the process. Ultrasound-guided transvaginal follicular aspiration was performed after 36 hours and 2 metaphase -II oocytes were collected, both of them were fertilized in vitro. However, only one cleavage-stage embryo with 5 cells was obtained on day 3. This embryo was then transplanted, but did not result in pregnancy. Then, the second cycle was initiated on June 3, 2016, on day 2 of bleeding, with a daily administration of 225 IU/d of hMG. However, the follicles were dysplastic and the dosage of hMG was increased to 350 IU/d with no success, thus resulting in the cycle being canceled. Afterwards, the patient started taking HRT to maintain a regular menstrual cycle. Finally, the third cycle began on December 14, 2016 using a GnRH agonist long protocol. A single dose of 0.8 mg of triptorelin (Ferring GmbH, Germany) was administered on day 18 of HRT to induce pituitary downregulation. Following 14 days of pituitary downregulation, the patient’s serum hormone concentrations had dropped to FSH: 4.17 IU/L, LH: 2.45 IU/L, and E2: 15.35 pg/mL. COS was then initiated with 150 IU/d of recombinant FSH (Gonal-f Merck Serono, Darmstadt, Germany) and 150 IU/d of hMG for 18 days. 75 IU/d of Luveris (Merck Serono, Darmstadt, Germany) was given for 6 days because a low LH level. Subsequently, when serum E2 was 3015 pg/mL and there were 8 follicles with a diameter ≥12 mm, 8000 IU of HCG was used to trigger ovulation and follicular aspiration was performed 36 hours later. A total of 8 Metaphase–II (MII) oocytes were collected and fertilized in vitro, yielding 5 cleavage-stage embryos on day 3, all embryos were vitrified and cryopreserved to prevent ovarian hyperstimulation syndrome. Two embryos were transplanted on May 14, 2017.Serum value of β-hCG was 1234 mIU/mL at 14th day after the transfer, and vaginal ultrasonography showed clinical twin pregnancy 28 days later. During the gestation period, there are no complications until the 33rd week. As a result of premature membrane rupture, the patient underwent a Caesarean section to deliver twin boys; both were in good health.

**Table 2:** Cycle characteristics and results in IVF.

Patient	Cycle	Protocol	Hormone levels when Gn started			Initial dose of Gn		Total Gn (Unit)	Days of stimulation	Serum E2 on triggering day (pg/mL)	Egg	MII	D3 embryo	The result
			FSH (IU/L)	LH (IU/L)	E2 (pg/mL)	r-FSH (Unit)	HMG (Unit)							
Case 1	1	Antagonist	18.58	10.71	79.98	–	225	3675	14	1479	2	2	1	Not pregnant
	2	Antagonist	13.34	7.25	55.21	–	225	4575	17	835	–	–	–	Cycle canceled
	3	GnRH agonist long protocol	4.17	2.45	15.35	150	150	5850	18	3015	8	8	5	Live birth
Case 2	1	GnRH agonist long protocol	7.79	7.31	19.14	150	150	2700	11	5934	27	23	10	Live birth

Note: E2 = estradiol, FSH = follicle-stimulating hormone, Gn = gonadotropin, GnRH = gonadotropin releasing hormone, HMG = human menopausal gonadotropin, IVF = in vitro fertilization, LH = luteinizing hormone, MII = metaphase–II, r-FSH = recombinant follicle-stimulating hormone.

### 2.2. Case 2

In June 2020, a 25-year-old nulliparous woman with secondary infertility for 3 years was referred to our IVF center. She had a history of ectopic pregnancy in 2017, and in total, experienced 6 years of infertility before the ectopic pregnancy. Her menarche occurred at age 14, with menstrual cycles ranging from 60 to 90 days. She reported no other medical conditions. Her baseline endocrine evaluation (Table 1) showed persistently elevated FSH levels across multiple menstrual cycles, while her AMH level remained within the normal range. An ultrasound scan revealed bilateral polycystic changes in her ovaries, with a total of over 24 antral follicles. Based on the treatment approach used for a previous patient, we opted for the GnRH agonist long protocol for COS. The patient underwent 3 cycles of hormonal pretreatment. On the 20th day of the third HRT cycle, she received a single dose of 0.7 mg triptorelin (Ferring GmbH, Germany) for pituitary downregulation. After 14 days, her serum hormone levels decreased as follows: FSH: 7.79 IU/L, LH: 7.31 IU/L, and E2: 19.14 pg/mL. Subsequently, she was administered 150 IU of recombinant FSH (Changchun Jinsai Pharmaceuticals, China) combined with 150 IU of hMG (Lizho Pharmaceuticals) to stimulate follicular growth. At day 11 of COS, 22 follicles with a diameter ≥12 mm were documented by ultrasonography and serum E2 was 5934 ng/mL. Ovulation was induced with injection of 8000 IU HCG. Ultrasound-guided transvaginal follicular aspiration was performed after 36 hours and 23 MII oocytes were collected. After in vitro fertilization, 4 cleavage-stage embryos were vitrified and cryopreserved and the remaining 6 embryos were further cultured, finally 2 blastocysts were cryopreserved. She achieved a live birth following a frozen-thawed embryo transfer with 1 blastocyst. Detailed information about her cycle characteristics and outcomes can be found in Table 2.

## 3. Discussions

### 3.1. Diagnosis and etiology of resistant ovary syndrome

ROS is relatively uncommon in clinical practice, accounting for approximately 11% to 20% of patients with hypergonadotropic amenorrhea.^[26]^ The primary clinical features of ROS include elevated gonadotropin levels and a normal ovarian reserve, which often leads to misdiagnosis as POF. The key distinction between ROS and POF is that ROS presents with a normal number of sinus follicles, while POF displays a few or none. In the past, the diagnosis of ROS relied on invasive ovarian biopsies to confirm the presence of sinus follicles.^[1]^ However, modern diagnostic methods have shifted towards noninvasive approaches, such as high-resolution transvaginal ultrasounds.^[27]^ Hormone biomarkers, including AMH and inhibin B, are now considered valuable diagnostic tools for ROS.^[2,28]^ These biomarkers are primarily secreted by granulosa cells and are strongly associated with ovarian reserve. Consequently, if a patient’s initial blood test reveals elevated gonadotropin levels, additional examinations for antral follicle count and AMH or inhibin B levels should be conducted to confirm a ROS diagnosis. The pathogenesis of ROS remains poorly understood, although early research suggested a possible link to immune factors. In 1982, researchers discovered that the serum of ROS patients with myasthenia gravis contained active substances that bind to FSH receptors, resulting in a lack of ovarian response to gonadotropin stimulation.^[29]^ Subsequent studies have provided further evidence supporting this hypothesis.^[20,21,30,31]^ Additionally, mutations in the FSH receptor gene have also been implicated as a potential cause of ROS.^[15,32,33]^

### 3.2. Resistant ovary syndrome treatment and literature review

Treatment for patients with ROS primarily targets the symptoms and long-term complications of estrogen deficiency while also assisting patients in achieving fertility when desired. HRT serves as the fundamental treatment for ROS patients, not only maintaining regular menstruation but also enabling some patients to conceive naturally. Research has shown that approximately 13% of ROS patients can achieve natural conception following low-dose HRT. This mechanism may be related to the inhibition of endogenous gonadotropin secretion, thereby increasing the sensitivity of granulosa cells.^[34]^ However, the majority of patients still require assisted reproductive technology, with conventional in vitro fertilization (c-IVF) typically being the first choice due to its established success and affordability. The critical factor for c-IVF success is a patient’s ovaries responding well to gonadotropin stimulation, resulting in a sufficient number of mature oocytes. Unfortunately, many patients fail to respond to gonadotropin, making it challenging to obtain mature oocytes. Currently, there is no clear consensus on COS protocols for ROS patients in clinical practice, leading physicians to often employ various ovulation induction schemes empirically. We reviewed COS protocols for ROS patients in IVF cycles through PubMed and WanFang databases from June 1969 to June 2023 (Table 3) and discovered that successful cases primarily focused on pituitary downregulation or the combined use of immunosuppressants.^[17,20,21]^ However, due to the heterogeneity of the underlying causes, the therapeutic effects of pituitary downregulation vary significantly. Therefore, determining how to correctly apply this protocol and identify the appropriate patient population will be the focus of future research.

**Table 3:** Summary of previous case reports on ROS patients undergoing COS.

Author	Age	BMI (kg/m^2^)	AFC	AMH (ng/mL)	Basal FSH (IU/L)	Hormonal pretreatment	Pituitary downregulation	Initial dose of Gn		Days of stimulation	Response to ovarian stimulation	MII eggs	Available embryo after c-IVF	Result
								r-FSH (Unit)	HMG (Unit)					
E. Koumantaki S., et al (1997)^[23]^	25	NA	NA	NA	27–67	NA	Yes	–	NA	15	no	–	–	Cycle canceled
Grynberg, M., et al (2013)^[13]^	29	Normal	18–23	4.5	38.4–40.3	NA	No	300	–	10	no	–	–	Cycle canceled
Rogenhofer, N., et al (2015)^[20]^	26	22	15	2.1	50.8	Estradiol and levonogestrel for 3 mo	Yes	75	225	14	yes	11	Two blastocysts	Pregnancy
Li, Y., et al (2016)^[3]^	33	Normal	25	12.27	41.99	Oral contraceptive Pills for current cycle	Yes	100	75	15	no	–	–	Cycle canceled
Flageole C., et al (2019)^[15]^	31	NA	19	3.24	55	oral contraceptive Pills for current cycle	Yes	–	300	16	no	–	–	Cycle canceled
Yang R., et al (2020)^[17]^	33	NA	19	2.47	18.4	NA	Yes	225	–	15	yes	12	9	Pregnancy
Alamtaj S., et al (2020)^[36]^	27	19.8	NA	2.46	29.5	NA	No	–	450	10	no	–	–	Cycle canceled
Huiying L., et al (2022)^[21]^	30	22.5	>20	6.29	42.37	NA	Yes	225	150	11	yes	8	3	Pregnancy
Fan Z., et al (2023)^[22]^	28–37 (6 patients)	17.6–24.1	7–17	1.46–5.71	19.11–46.95	NA	Yes (6 cycles)	NA	NA	NA	yes	12	8	Two patients pregnancy
						NA	No (10 cycles)	NA	NA	NA	no	0	0	Cycle canceled

*Note*. NA = not applicable.

### 3.3. Reasons analysis for the success of 2 cases

In this report, we present 2 successful cases utilizing the pituitary downregulation protocol. These patients exhibited a favorable response to this protocol, which may be attributed to the following factors:

First, both patients demonstrated a mild to moderate increase in FSH levels (<40 IU/L), suggesting that their ovarian resistance to gonadotropin might not be severe. Previous research has proposed that different basal FSH levels can reflect the degree of ovarian resistance to gonadotropin (Gn), with higher FSH levels indicating more severe Gn resistance, particularly when FSH exceeds 40 IU/L.^[35]^ Subsequent studies have confirmed this view, suggesting that ROS patients with basal FSH levels over 40 IU/L are more likely to exhibit poor or no response to exogenous gonadotropin.^[17]^

Second, pituitary downregulation can effectively inhibit the secretion of endogenous FSH and LH, increasing ovarian sensitivity to exogenous Gn. Additionally, 3 cycles of HRT pretreatment may benefit the upregulation of FSH receptors in granulosa cells and downregulation of endogenous gonadotropins. Rogenhofe et al proposed that 3 cycles of HRT pretreatment may contribute to successful treatment outcomes.^[20]^ Although we did not continuously monitor gonadotropin titers to confirm this, Andreas Mueller et al observed a significant decrease in serum LH and FSH levels in ROS patients during the third month of HRT, accompanied by increased estradiol, progesterone, and Inhibin A levels, indicating spontaneous follicular growth and maturation.^[10]^ This observation may explain why some patients resume normal ovulation after a period of HRT and eventually conceive naturally.

Third, selecting the appropriate ovarian stimulation drugs is crucial for follicular development in ROS patients. Clinically, human menopausal gonadotropin (HMG) is often used or recommended for ovulation induction in ROS patients.^[3,15,20,21,23,35,36]^ HMG’s primary components are FSH and LH, with LH playing a vital physiological role in follicular steroid hormone production and development. Researchers have suggested that gene mutation of rs2293275 may result in decreased LH receptor sensitivity, increasing in vivo LH levels to enhance LH effects and upregulate FSH receptor expression, thereby improving ovarian reactivity in ROS patients.^[36]^ Rogenhofer’s study found that ROS patients had specific antibodies bound to HMG, but not to r-FSH. As a result, we selected r-FSH combined with HMG for COS to maximize the immunological diversity coverage of FSH and LH isoforms.^[20]^ For the first patient, we used HMG alone during the initial 2 cycles, resulting in a poor ovarian response. In the third cycle, we combined r-FSH, leading to a favorable response. Unfortunately, we were unable to detect FSH antibodies in patients.

Finally, high-dose gonadotropin priming overcomes gonadotropin resistance as much as possible, reaching or even exceeding the FSH threshold required for follicle development. In these 2 cases, the initial gonadotropin dose reached 300 units per day.

## 4. Limitations

Our study acknowledges the constraints that may affect the generalizability of our findings. Primarily, the inherently low incidence of ROS restricts our ability to accumulate a large sample size and, consequently, may limit the statistical power of our conclusions. Moreover, the retrospective nature of our study introduces the possibility of selection bias and reduces the ability to establish causal relationships. We propose that further research, ideally through prospective studies with larger cohorts, be conducted to substantiate and refine the effectiveness of the COS protocol presented.

## 5. Conclusions

In conclusion, ROS presents a significant challenge in managing reproductive endocrine disorders. C-IVF remains a cost-effective approach for achieving successful pregnancies in ROS patients. Implementing a pituitary downregulation protocol using recombinant follitropin in combination with HMG, following a minimum of 3 HRT pretreatment cycles, may prove beneficial.

## Acknowledgments

The authors would like to thank the participant in this study.

## Author contributions

**Conceptualization:** Shanfei Zhao, Guanyun Long.

**Data curation:** Wenling Zheng, Xinru Gu.

**Formal analysis:** Shanfei Zhao, Wenling Zheng, Guanglin Liang.

**Investigation:** Shanfei Zhao, Xinru Gu, Guanglin Liang.

**Methodology:** Shanfei Zhao, Guanyun Long.

**Writing – original draft:** Shanfei Zhao.

**Writing – review & editing:** Shanfei Zhao, Guanyun Long.
